# Angiotensin-converting enzyme 2 in dogs with *Dirofilaria immitis*

**DOI:** 10.1186/s13071-023-05649-9

**Published:** 2023-04-28

**Authors:** Darcy B. Adin, Meaghan Spalla, Heather Walden, Jeff Gruntmeir, Jorge A. Hernandez, Maureen Long

**Affiliations:** College of Veterinary Medicine, University of Florida, Gainesville, FL USA

**Keywords:** Heartworm, RAAS, Weight, Inflammation, Cardiopulmonary disease

## Abstract

**Background:**

Infection by the canine heartworm, *Dirofilaria immitis*, causes significant cardiopulmonary disease, with progression impacted by increasing parasite numbers and duration of infection. The renin–angiotensin–aldosterone system (RAAS) is an important mediator of cardiac and pulmonary disease. Angiotensin-converting enzyme 2 (ACE2) mitigates the maladaptive effects of angiotensin II by converting it to angiotensin (1-7). We hypothesized that circulating ACE2 activity would be altered in dogs with high heartworm infection intensities relative to dogs without heartworms.

**Methods:**

Frozen serum samples (−80 °C) from 30 dogs euthanized at Florida shelters were analyzed for ACE2 activity using liquid chromatography–mass spectrometry/mass spectroscopy and a kinetics approach with and without an ACE2 inhibitor. A convenience sample of 15 dogs without heartworms (HW_0_) and 15 dogs with > 50 heartworms (HW_>50_) was included. Heartworm number and microfilariae presence were determined at necropsy. The effects of heartworm status, body weight, and sex on ACE2 were evaluated using regression analysis. Values of *P* < 0.05 were considered significant.

**Results:**

All HW_0_ dogs were *D. immitis* microfilariae-negative and all HW_>50_ dogs were *D. immitis* microfilariae-positive with a median adult worm count of 74 (minimum = 63, maximum = 137). The ACE2 activity of HW_>50_ dogs (median = 28.2 ng/ml; minimum = 13.6, maximum = 76.2) was not different from HW_0_ dogs (median 31.9 ng/ml; minimum = 14.1, maximum = 139.1; *P* = 0.53). The ACE2 activity was higher in dogs with high body weight (median 34.2 ng/ml minimum = 14.1, maximum = 76.2) than in dogs with low weight (median 27.5 ng/ml; minimum = 16.4, maximum = 139.1; *P* = .044).

**Conclusions:**

Heartworm infection did not impact ACE2 activity in shelter dogs with or without heartworms, but heavier dogs had higher ACE2 activity compared to lighter dogs. Comprehensive RAAS evaluation and additional clinical information would aid in understanding how ACE2 activity relates to the entire cascade and clinical status in dogs with heartworm disease.

**Graphical Abstract:**

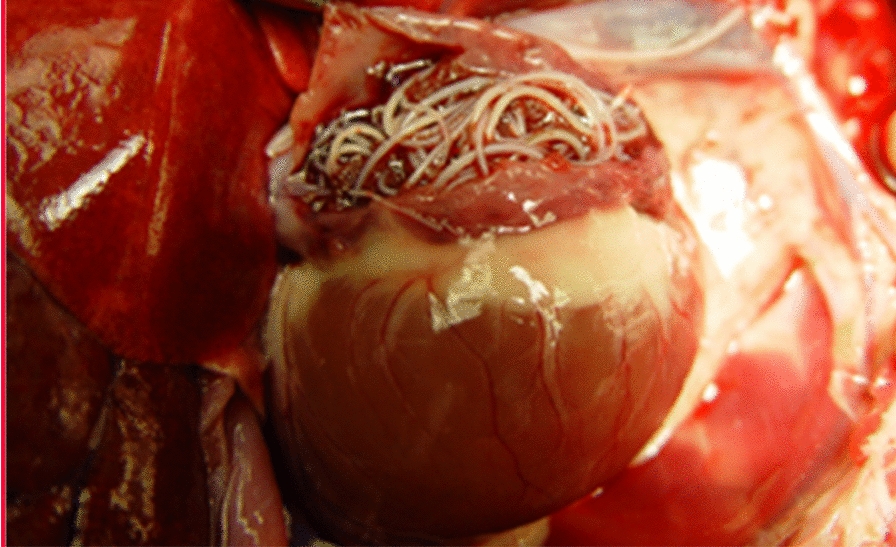

**Supplementary Information:**

The online version contains supplementary material available at 10.1186/s13071-023-05649-9.

## Background

Heartworm disease caused by *Dirofilaria immitis* infection is endemic in the canine population despite the availability of preventive medications [1]. *Dirofilaria immitis* causes pulmonary pathology that can lead to cardiac failure if not treated [2]. The consequences of adult worm residence in the pulmonary arteries include pulmonary vascular endarteritis, pulmonary parenchymal inflammation, pulmonary thromboembolism, and pulmonary hypertension, leading to clinical signs of cough, shortness of breath, collapse, and congestive heart failure [1–5]. Cardiopulmonary pathology can persist after successful worm eradication [5].

Increased inflammatory mediators such as acute phase proteins and markers of oxidative stress predict disease severity and have aided the understanding of heartworm disease pathogenesis [6–8]. Additionally, selected renin–angiotensin–aldosterone system (RAAS) components, including renin, angiotensin II, and atrial natriuretic peptide, are higher in dogs with severe heartworm disease than in healthy dogs, supporting a role for the RAAS in advanced disease with heart failure [9]. The RAAS is an important neurohormonal regulator of cardiac, renal, and vascular function as well as inflammation [10]. Because of the vasoconstriction, inflammation, and fluid retention enacted by mediators such as angiotensin II and aldosterone, the RAAS has classically been viewed as maladaptive, promoting dysfunction and progression of heart failure. New research has elucidated the role of an alternative RAAS pathway mediated by the Mas receptor that counterbalances maladaptive effects through vasodilation, bradycardia, natriuresis, and anti-inflammation [10]. The transmembrane protein angiotensin-converting enzyme 2 (ACE2) is a catalyst for the alternative (beneficial) RAAS pathway in that it metabolizes angiotensin II into angiotensin (1-7), which then interacts with the Mas receptor to cause these beneficial physiological effects [11].

The protective effects of ACE2, and the resulting production of alternative RAAS metabolites, are not limited to cardiac diseases, but also extend to lung injury, where the alternative RAAS pathway facilitates vasodilatory effects through angiotensin (1-7) [12]. The transmembrane protein ACE2 is expressed in lung tissue and is protective regardless of pulmonary pathology etiology. Recently, significant attention has been paid to its role in the pandemic caused by the novel coronavirus (SARS-CoV-2) [13, 14]. This virus uses ACE2 as a receptor for cell entry and in doing so, causes ACE2 downregulation, allowing for unopposed angiotensin II effects [12, 15–17]. Lower baseline ACE2 levels, or ACE2 internalization after interaction with SARS-CoV-2 and other viruses, contribute to lung damage associated with infections and inflammation [18, 19]. Recombinant ACE2 administration, however, can bind viral particles, thereby benefiting patients with severe lung disease caused by SARS-CoV-2 [12, 20]. Conversely, naturally higher soluble ACE2 activity in disease states predicts mortality, possibly as a result of upregulation or shedding of the membrane-bound form of ACE2 [13]. Research surrounding the role of ACE2 in SARS-CoV2 infection highlights the complexity of ACE2 related to disease pathology.

The RAAS has only been investigated in dogs with advanced heartworm disease and heart failure, and no studies have evaluated ACE2 in this population [9]. The role of ACE2 in heartworm-related pulmonary pathology is unexplored and could be complex. The objective of this study was to determine whether ACE2 activity is different in dogs with high heartworm burden relative to heartworm-free dogs. We hypothesized that circulating (soluble) ACE2 activity would be altered in dogs with heavy heartworm burden because of attendant pulmonary pathology, relative to dogs free of heartworms.

## Methods

A convenience sample of 30 euthanized dogs with (*n* = 15) or without (*n* = 15) heartworms at Florida shelters was included. Frozen serum (−80 °C) samples from the 30 study dogs were analyzed for ACE2 activity using liquid chromatography-mass spectrometry/mass-spectroscopy and an equilibrium dialysis, kinetics approach utilizing a spiked substrate, with and without an ACE2 inhibitor (Attoquant Diagnostics). The dogs were euthanized for reasons determined by the shelter and not for the purpose of this study. The time from euthanasia to sample collection was approximately 2 h, and samples were immediately processed and frozen. Sample collection was approved by the Institutional Animal Care and Use Committee at the University of Florida (protocol #202010115). Sex, body weight, heartworm number, and microfilariae presence were determined at necropsy when blood was collected. Dogs were included in the heartworm-positive group if they had more than 50 adult heartworms identified at necropsy (HW_>50_) and in the heartworm negative group if they had no adult heartworms identified at necropsy (HW_0_).

Data for ACE2 activity and body weight were calculated and reported as mean ± SD as well as median, minimum, and maximum values. Dogs were assigned to one of two body weight groups (low: 4–23 kg, *n* = 17; high = 24–37 kg, *n* = 13) based on the median distribution. The ACE2 activity was compared between HW_0_ dogs and HW_>50_ dogs, and between dogs with high or low body weight using the non-parametric Wilcoxon rank-sum test. Multiple linear regression was used to compare circulating ACE2 activity (rank data) between HW_0_ dogs and HW_>50_ dogs after controlling for body weight group or sex. In addition, multiple linear regression was used to compare ACE2 activity between dogs with high and low body weight, after controlling for diagnosis of heartworm infection or sex. Finally, the relationship between sex or body weight group and diagnosis of heartworm infection was tested using a Chi-square test. Values of *P* < 0.05 were considered statistically significant.

## Results

There were nine intact female and six intact male dogs in the HW_0_ group. There were six intact female and nine intact male dogs in the HW_>50_ group. All HW_0_ dogs were *D. immitis* microfilaria-negative and all HW_>50_ dogs were *D. immitis* microfilariae-positive and had a median adult worm count of 74 (minimum = 63, maximum = 137). The variables for body weight and ACE2 activity were not normally distributed. The median body weight of all 30 dogs was 22.7 kg. There was no weight difference (Wilcoxon rank-sum test, *Z* = 0.38, *P* = 0.70) or sex difference (*χ*^2^ = 1.20; DF = 1; *P* = 0.27) between groups (Table 1). Additional file 1: Dataset 1 shows data from all dogs.

**Table 1:** ACE2 activity, body weight, and sex of dogs without heartworms (HW_0_) and dogs with more than 50 heartworms (HW_>50_)

	HW_0_ *n* = 15	HW_>50_ *n* = 15	*P*
ACE2 activity (ng/ml)^a^	39.2 ± 29.3 31.9 (14.1, 139.1)	32.6 ± 15.4 28.2 (16.4, 76.2)	0.53
Body weight (kg)^a^	20.9 ± 11.5 22.7 (4.5, 36.3)	23.3 ± 6.8 22.7 (13.6, 36.3)	0.7
Body weight^b^			0.71
Low: 4.5 to 22.7 kg	9	8	
High: 27.2 to 36.3 kg	6	7	
Sex^b^			0.27
Female	9	6	
Male	6	9	

^a^Data are reported as mean ± SD and median (minimum, maximum)

^b^Data are reported as observed frequencies, *n*

The ACE2 activity of HW_>50_ dogs (median = 28.2 ng/ml; minimum = 16.4, maximum = 76.2) was not different from HW_0_ dogs (31.9 ng/ml; 14.1, 139.1; Wilcoxon rank-sum test, *Z* = 0.62, *P* = 0.53; Table 1).

The ACE2 activity was higher in dogs with high body weight (median = 34.2 ng/ml; minimum = 14.2, maximum = 76.2) compared to those with low body weight (median 27.5 ng/ml; minimum = 16.4, maximum = 139.1) (Wilcoxon rank sum test, *Z* = 2.01, *P* = 0.044).

Using multiple linear regression analysis, ACE2 activity was not different between HW_>50_ and HW_0_ dogs, after controlling for low/high body weight group (*P* = 0.42) or sex (*P* = 0.41). In addition, dogs with high body weight had higher ACE2 activity than those with low body weight, after controlling for diagnosis of heartworm infection (*P* = 0.037) or sex (*P* = 0.026).

## Discussion

We did not find differences in circulating (soluble) ACE2 activity in this cohort of shelter dogs when dogs were grouped by the presence or absence of necropsy-confirmed adult heartworms. These results do not support a role for this enzyme as a biomarker of heartworm disease, but the results should be interpreted in light of the study population and design. Although we used serum from dogs with a high number of intracardiac heartworms identified at necropsy to target a population with severe heartworm disease, the clinical status, and therefore the effect of the worms on cardiopulmonary structure and function, was not known. Likewise, the HW_0_ dogs could have had other cardiopulmonary diseases which might have influenced ACE2 activity. The activity of membrane-bound (tissue) ACE2 might be altered differently from soluble ACE2 activity as a result of up- or downregulation or disease-induced shedding of the membrane-bound protein. The lack of differences between HW_0_ dogs and HW_>50_ dogs in this study, however, do not support the presence of active shedding of membrane-bound ACE2 with release into the circulation or upregulation of soluble ACE2 in dogs with heavy heartworm burden.

A previous study demonstrated elevations in renin, angiotensin II, and atrial natriuretic peptide in dogs with advanced heartworm disease characterized by congestive heart failure or caval syndrome [9]. Since congestive heart failure is associated with RAAS activation, it is unclear whether the high metabolite concentrations in this study were due to heartworm disease, congestive heart failure, or both. Clinical status was not available for the dogs in our study, and we measured a different RAAS component than the previous study, making the findings not directly comparable.

Although the study sample was small, the results revealed a positive relationship between body weight and circulating ACE2 activity in study dogs. The reason for higher ACE2 activity in heavier dogs is not known; however, a similar direct relationship between ACE2 activity and body mass index was recently reported in boys [21]. Additionally, a study in dogs reported an inverse relationship between the urinary aldosterone-to-creatinine ratio (a marker of classical RAAS activation) and body weight, which is consistent with our results, as ACE2 is a marker of the alternative RAAS [22]. In that previous study, the authors showed that lighter dogs had higher urinary aldosterone, and in our study, we showed that lighter dogs had lower ACE2 activity, both of which are consistent with more classical RAAS influence than alternative RAAS influence in dogs with low body weight [22]. Unidentified factors associated with both ACE2 activity and body weight might have affected our results, especially considering the limited clinical data available for the study dogs. Numerous modifiers of RAAS expression are known in humans and in dogs [23–26], and so it is plausible that measures of body mass could also affect ACE2. Our results indicate that body weight should be considered in studies evaluating the RAAS.

The ACE2 activity for all dogs in our study was 35 times higher than that reported in healthy people and 17 times higher than that reported in people with cardiopulmonary diseases using the same methodology [27]. Species differences in other aspects of the RAAS profile are evident from previous studies 28, 29, but reports of ACE2 by equilibrium analysis are limited. Our results support species differences in soluble ACE2, the predominant enzyme of the alternative RAAS cascade. Higher ACE2 activity might reflect different physiological adaptations or different genetic influences of dogs compared with humans.

The results of our study provide initial insight into the RAAS of dogs with heartworm infection using the relatively new approach of equilibrium dialysis, but there are several limitations to consider for interpretation. The clinicopathological characterization of HW_0_ and HW_>50_ dogs was limited, and therefore it is possible that some dogs had confounding conditions that could have impacted ACE2 activity. Additionally, the presence of a large number of heartworms does not definitely mean that those dogs had cardiopulmonary pathology associated with heartworm residence in the pulmonary vasculature and the duration of infection, which was unknown for these dogs, could be a contributing factor in disease progression. The body condition score was not available for included dogs, and this omission might have affected the evaluation of the effect of body weight on ACE2 activity, especially if there were dogs in the study with very high or very low body condition scores. We only measured circulating (soluble) ACE2 activity, and it is possible that measurement of the membrane-bound (tissue) form would have yielded different results. Because membrane-bound ACE2 might be more important for cardiopulmonary protection, it should be investigated in future studies despite the negative results for soluble ACE2. The small sample size limited the weight divisions to those under and over the median weight for the groups, and a larger study would be needed to refine the assessment of the effect of weight on ACE2 activity. Lastly, it is possible that the remainder of the RAAS cascade could differ between these heartworm-infected and non-infected dogs because of the many influences of the formation and degradation of other metabolites and the relative balance between enzymes.

## Conclusions

Our study did not support a role for soluble ACE2 in dogs with heavy heartworm burden, but we found that dogs with high body weight had higher ACE2 activity than dogs with low body weight, supporting weight as a potential confounding factor in the evaluation of ACE2. We also found that ACE2 activity in our sample of study dogs was higher than that reported in people, supporting species differences. The lack of soluble ACE2 differences between dogs with and without heartworm infection does not mean that differences in membrane-bound ACE2 or other components of the RAAS cascade are not present in dogs with heartworm infection. Comprehensive RAAS evaluation including all known metabolites and other enzymes, combined with additional clinical information, would provide insight into metabolite relationships to understand how ACE2 activity relates to the entire RAAS cascade and clinical status in dogs with heartworm disease.

### Supplementary Information

**Additional file 1.** Dataset of individual dog results.

## Data Availability

Raw data are available in the Additional file 1.

## Abbreviations

ACE2: Angiotensin-converting enzyme 2
HW: Heartworm
RAAS: Renin-angiotensin-aldosterone system
